# Forelimb bone curvature in terrestrial and arboreal mammals

**DOI:** 10.7717/peerj.3229

**Published:** 2017-04-26

**Authors:** Keith Henderson, Jess Pantinople, Kyle McCabe, Hazel L. Richards, Nick Milne

**Affiliations:** School of Anatomy, Physiology and Human Biology, University of Western Australia, Perth, Western Australia, Australia

**Keywords:** Humerus, Ulna, Curvature, Arboreal, Terrestrial, Adaptation

## Abstract

It has recently been proposed that the caudal curvature (concave caudal side) observed in the radioulna of terrestrial quadrupeds is an adaptation to the habitual action of the triceps muscle which causes cranial bending strains (compression on cranial side). The caudal curvature is proposed to be adaptive because longitudinal loading induces caudal bending strains (increased compression on the caudal side), and these opposing bending strains counteract each other leaving the radioulna less strained. If this is true for terrestrial quadrupeds, where triceps is required for habitual elbow extension, then we might expect that in arboreal species, where brachialis is habitually required to maintain elbow flexion, the radioulna should instead be cranially curved. This study measures sagittal curvature of the ulna in a range of terrestrial and arboreal primates and marsupials, and finds that their ulnae are curved in opposite directions in these two locomotor categories. This study also examines sagittal curvature in the humerus in the same species, and finds differences that can be attributed to similar adaptations: the bone is curved to counter the habitual muscle action required by the animal’s lifestyle, the difference being mainly in the distal part of the humerus, where arboreal animals tend have a cranial concavity, thought to be in response the carpal and digital muscles that pull cranially on the distal humerus.

## Introduction

The presence of curvature in mammalian limb bones appears biomechanically paradoxical, and so has been the subject of research and discussion for over 50 years. Curved bones are weaker under longitudinal loading (Biewener, 1983; Lanyon & Rubin, 1985; Jade et al., 2014), and in many situations remodel to become straight (Frost, 1964). However, many long bones in animal limbs maintain a degree of curvature (Lanyon, 1980; Biewener, 1983; Swartz, 1990; Drapeau et al., 2005; Jade et al., 2014). Probably the most-studied curved bone is the radius of obligate quadruped species like sheep, goats and horses (Bertram & Biewener, 1992; Lanyon, Magee & Baggott, 1979). These bones have a concavity on the caudal side (caudal curvature) and strain gauge studies have demonstrated (Lanyon & Baggott, 1976; Lanyon, Magee & Baggott, 1979) that during stance phase the radius is indeed subjected to strains that increase the existing curvature (caudal bending). However in another study, this time involving the tibia, the strains during stance phase were in the opposite direction, tending instead to reduce curvature (Lanyon & Bourn, 1976). It has been hypothesised that bone curvature exists to accommodate bulky musculature (Lanyon, 1980; Swartz, 1990), that it increases bending strains to stimulate remodelling and improve bone strength (Lanyon, 1980), or it may provide an early warning if bones approach their loading limits (Currey, 1984). Bertram & Biewener (1988) suggested that curved bones benefit from the predictable bending that results from curvature, but these authors did not provide examples of how soft tissue mechanisms might mitigate that predictable bending. Swartz (1990, p. 496) commented “…it is necessary to develop a way in which the concept of predictability can be made more explicitly operational”.

The radius and ulna are fused or tightly connected in obligate quadrupeds (Chauveau, 1890), and recently, Milne (2016) suggested that the habitual pull of triceps on the olecranon of the ulna could counter the predictable bending within the radioulnar shaft. He went on to demonstrate, using finite elements analysis, that a curved llama radioulna subjected to triceps pull and longitudinal and carpal flexor loads, is less stressed than a straight version of the same bone. He suggested that the curvature of the llama radioulna was an adaption to the habitual action of the triceps muscle, where the predictable caudal bending that results from bone curvature provides a mechanism to counter the cranial bending due the habitual action of triceps—the ‘curved bone effect’. While the example presented was the llama radioulna, Milne suggested the idea may apply to all terrestrial quadrupeds, where the action of triceps is necessary to maintain stance.

If this is true for terrestrial quadrupeds, then we might expect that in arboreal species, where elbow flexion must be maintained, forearm bones should instead be cranially curved. In terrestrial quadrupeds, the (radio) ulna is a lever dominated by the action of triceps maintaining stance (elbow extension) against gravity, and thus the bone develops a caudal curvature. However, in arboreal species the biceps and brachialis muscles are vital in maintaining elbow flexion, to grasp, cling and lift the animal among the branches. If the habitual action of triceps is the cause of the caudal curvature of the radioulna in terrestrial species, then we expect arboreal species, where the forearm is dominated by the habitual action of brachialis, to have a cranially curved (radio) ulna.

Marsupials and primates are two taxa of mammals in which some species are arboreal and some are terrestrial. On this basis, this study examines the curvature of the ulna in a wide range of marsupials and primates to test the hypothesis that terrestrial species have caudally curved ulnae, and arboreal species have cranially curved ulnae. Primates and marsupials were chosen because they are two unrelated groups of mammals, so if the same pattern of variation occurs in both then we can be confident that the observed variation is not related to common ancestry of arboreal or terrestrial quadrupeds.

If the ulna shows a different curvature in terrestrial and arboreal species, it raises the question: is this phenomenon limited to the ulna, or do other bones, like the humerus, respond to habitual loading in similar ways? It is difficult to propose an *a priori* hypothesis about the expected curvature of the humerus in response to extensile or prehensile lifestyles. However, in order to determine if the relationship between locomotor ecology to forelimb bone curvature applies more generally, we also examined the anteroposterior curvature of the humerus in relation to arboreal and terrestrial lifestyles.

## Materials and Methods

The sample included the ulna and humerus from a wide range of primate (*n* = 28) and marsupial (*n* = 42) species (see Tables 1 and 2). Only adult or semiadult specimens were examined. The sex of most of the specimens was unknown, and so was presumed to be mixed in all analyses.

**Table 1:** Primate species and mean curvature (and number) of ulnae and humeri used. Locomotor behaviour of each species is also shown. Locomotor behaviour is classified as 1, arboreal; 2, semi arboreal; 3, terrestrial.

Family	Primate species	Common name	Ulnae	Humeri	Locomotion
*Lorisidae*	*Nycticebus coucang*	Slow loris	−0.029 (3)	0.037 (3)	1
	*Nycticebus tardigradus*	Bengal loris	−0.033 (1)	0.04 (1)	1
	*Perodicticus potto*	Potto	−0.048 (1)	0.029 (1)	1
	*Galago sp.*	Bushbaby	0.02 (1)	0.035 (1)	1
*Lemuridae*	*Eulemur mongoz*	Mongoose lemur	−0.023 (2)	0.063 (3)	1
	*Lemur catta*	Ring-tailed lemur	−0.014 (2)	0.049 (2)	2
	*Varecia variegata*	Ruffled lemur	−0.034 (1)	0.064 (1)	2
*Indriidae*	*Indri indri*	Indri	−0.012 (1)	−0.028 (1)	1
	*Propithecus verreauxi*	Sifaka	−0.049 (2)	0.036 (2)	1
	*Propithecus diadema*	Sifaka	−0.054 (2)	0.033 (2)	1
*Callitrichidae*	*Leontropithecus rosalia*	Golden lion tamarin	−0.021 (1)	0.056 (1)	1
	*Callithrix jacchus*	Marmoset	0.033 (6)	0.05 (6)	1
*Cebidae*	*Cebus capucinus*	White-faced capuchin	0.028 (1)	0.05 (1)	1
	*Ateles sp.*	Spider monkey	0.003 (1)	0.067 (1)	1
*Cercopithidae*	*Macaca fascicularis*	Crab-eating macaque	−0.015 (4)	0.054 (3)	1
	*Macaca fuscatus*	Japanese macaque	−0.008 (1)	0.048 (1)	2
	*Macaca silenus*	Liontail macaque	−0.001 (2)	0.053 (2)	1
	*Macaca nemestrina*	Pigtail macaque	−0.016 (3)	0.051 (3)	2
	*Macaca nigra*	Celebes ape	0.007 (3)	0.064 (2)	2
	*Papio hamadryas*	Hamadryas baboon	0.011 (3)	0.064 (3)	3
	*Papio ursinus*	Chacma baboon	0.004 (3)	0.61 (3)	3
	*Mandrillus leucophaeus*	Drill	0.03 (3)	0.056 (3)	3
	*Trachypithecus pileatus*	Capped langur	−0.018 (1)	0.043 (1)	1
	*Procolobus verus*	Olive colobus	−0.018 (1)	0.041 (1)	1
	*Chlorocebus aethiops*	Grivet	−0.011 (1)	0.071 (1)	2
*Hominidae*	*Pongo pygmaeus*	Orangutan	−0.06 (4)	−0.01 (5)	1
	*Gorilla gorilla*	Gorilla	−0.031 (1)	−0.022 (1)	3
	*Pan troglodytes*	Chimpanzee	−0.044 (6)	−0.012 (4)	2

**Table 2:** Marsupial species and mean curvature (and number) of ulnae and humeri used. Locomotor behaviour of each species is also shown. Locomotor behaviour is classified as 1, arboreal; 2, semi arboreal; 3, terrestrial.

Family	Marsupial species	Common name	Ulna	Humerus	Locomotion
*Didelphidae*	*Didelphys virginiana*	Virginia opossum	0.012 (1)	0.083 (1)	1
*Thylacinidae*	*Thylocinus cynocephalus*	Tasmanian tiger	0.051 (1)	0.01 (4)	3
*Dasyuridae*	*Phascogale tapoatafa*	Brush-tailed phascogale	0.006 (2)	0.057 (2)	1
	*Dasyuroides byrnei*	Kowari	0.023 (2)	0.096 (2)	3
	*Dasycercus cristicuda*	Crest-tailed mulgara	0.018 (1)	0.121 (1)	3
	*Dasyurus hallacatus*	Northern quoll	0.011 (7)	0.081 (8)	3
	*Dasyurus viverrinus*	Eastern quoll	0.012 (7)	0.102 (9)	3
	*Dasyurus geoffroii*	Western quoll	0.023 (8)	0.101 (5)	3
	*Dasyurus albopunctatus*	New Guinean quoll	−0.008 (2)	0.107 (3)	2
	*Dasyurus maculatus*	Tiger quoll	−0.006 (20)	0.105 (21)	2
	*Sarcophilus harrisii*	Tasmanian devil	0.021 (11)	0.135 (9)	3
*Myrmecobiidae*	*Myrmecobius fasciatus*	Numbat	0.03 (1)	0.068 (1)	3
*Peramelidae*	*Perorycytes longicauda*	Striped bandicoot	0.024 (1)	0.067 (2)	3
	*Perameles nasuta*	Long-nosed bandicoot	0.021 (14)	0.091 (14)	3
	*Perameles gunnii*	Eastern barred bandicoot	0.028 (3)	0.117 (4)	3
	*Echymipera kalubu*	Common spiny bandicoot	−0.028 (2)	0.084 (2)	3
	*Isoodon macrourus*	Northern brown bandicoot	0.019 (3)	0.078 (3)	3
	*Isoodon obesulus*	Southern brown bandicoot	0.03 (3)	0.088 (5)	3
	*Isoodon auratus*	Golden bandicoot	0.037 (1)	0.082 (1)	3
*Thylacomyidae*	*Macrotis lagotis*	Greater bilby	−0.012 (1)	0.122 (4)	3
*Phascolarctidae*	*Phascolarctos cinerus*	Koala	−0.036 (10)	0.072 (8)	1
*Vombatidae*	*Vombatus ursinus*	Common wombat	0.015 (4)	0.092 (4)	3
	*Lasiorhinus latifrons*	Southern hairy-nosed wombat	−0.029 (4)	0.081 (3)	3
*Phalangeridae*	*Trichosurus vulpecula*	Common brushtail possum	−0.021 (10)	0.075 (16)	1
	*Trichosurus caninus*	Short-eared possum	−0.021 (2)	0.086 (3)	1
	*Phalanger orientalis*	Northern common cuscus	−0.038 (2)	0.092 (2)	1
	*Phalanger carmelitae*	Mountain cuscus	−0.028 (1)	0.085 (2)	1
	*Phalanger sericeus*	Silky cuscus	−0.022 (1)	0.069 (1)	1
	*Phalanger intercastellanus*	Eastern common cuscus	−0.029 (2)	0.083 (2)	1
	*Phalanger maculatus*	Common spotted cuscus	−0.042 (1)	0.061 (2)	1
	*Phalanger gymnotis*	Ground cuscus	−0.054 (1)	0.084 (1)	3
	*Gymnobelideus leadbeateri*	Leadbeater’s possum	−0.032 (1)	0.094 (1)	1
*Petauridae*	*Petaurus norfolcensis*	Squirrel glider	−0.013 (12)	0.067 (12)	1
	*Petaurus australis*	Yellow-bellied glider	−0.028 (2)	0.06 (2)	1
	*Petauroides volans*	Greater glider	0.011 (8)	0.054 (9)	1
	*Pseudocheirus peregrinus*	Common ringtail possum	−0.042 (1)	0.08 (1)	1
	*Pseudocheirus occidentalis*	Western ringtailed possum	−0.031 (2)	0.073 (4)	1
	*Dactylopsila trivigata*	Striped possum	−0.022 (1)	0.083 (5)	1
	*Dactylopsila magalura*	Great-tailed triok		0.08 (3)	1
*Macropodidae*	*Dendrolagus lumholtzi*	Lumholtz’s tree-kangaroo	−0.038 (3)	0.065 (4)	1
	*Dendrolagus bennettianus*	Bennett’s tree-kangaroo	−0.036 (1)		1
	*Dendrolagus matschiei*	Matschie’s tree-kangaroo	−0.034 (1)	0.052 (1)	1

Specimens were determined to be arboreal, semiarboreal or terrestrial (Nowak, 1991; Grueter et al., 2013). Arboreal species live in trees and use their forelimbs in flexion to raise themselves and cling to branches. Terrestrial species are quadrupeds where triceps likely dominate forelimb function; if they do climb they do so by walking on top of branches. Semiarboreal species may spend most of their time on the ground, but are adept at climbing (quolls), or conversely, may be mostly arboreal but also spend much time on the ground (most semiarboreal primates).

**Figure 1:**
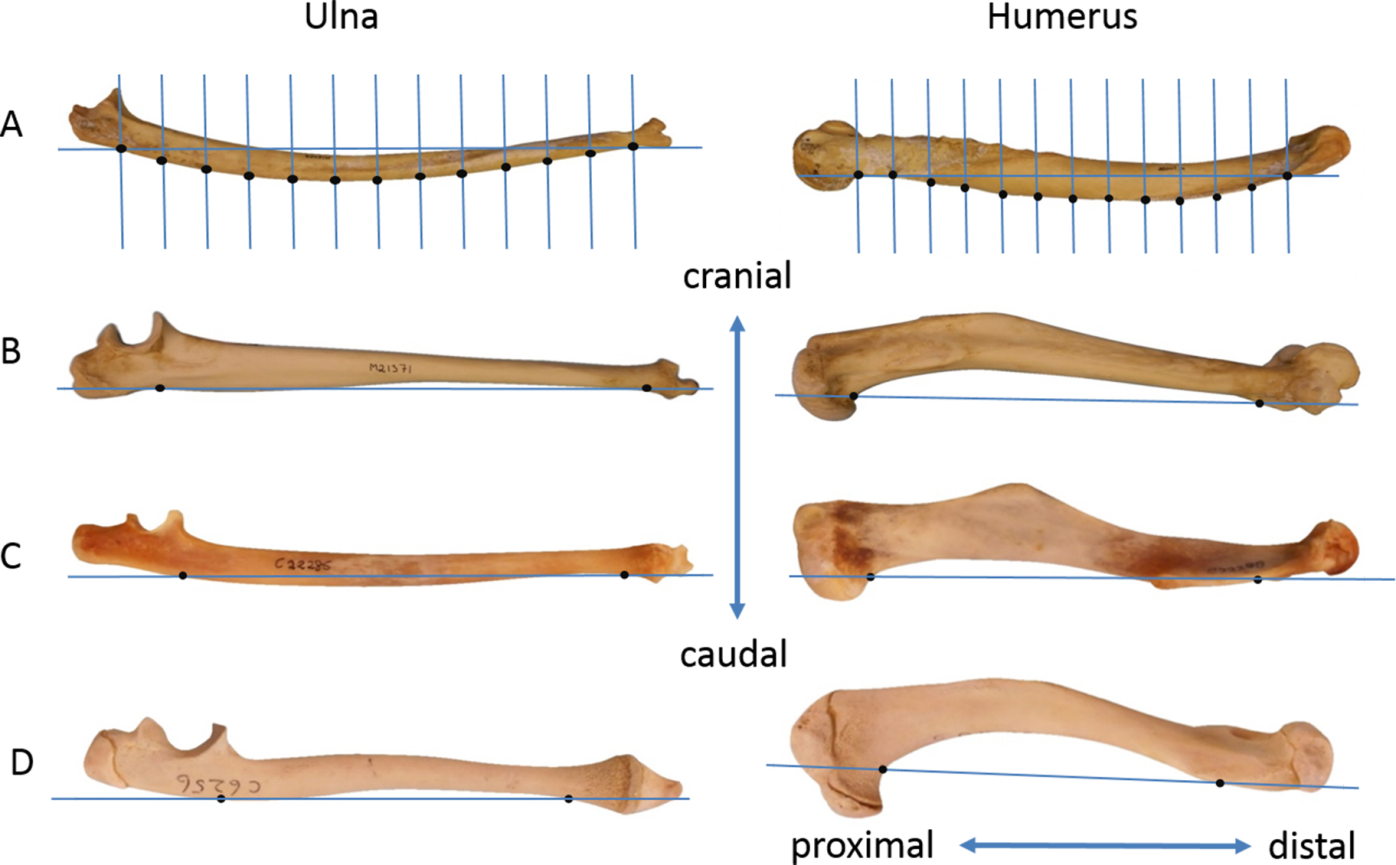
Lateral photographs of some ulnae and humeri. (A) *Pongo pygmaeus* ulna and humerus with the chord and 13 parallel lines together with the constructed landmarks. (B) *Papio hamadryas*, (C) *Phascolarctos cinereus*, (D) *Sarcophilus harrisii*. In parts (B), (C) and (D) only the chord and its endpoints are marked.

The ulna and humerus of each specimen were photographed in a standardised orientation that captured the curvature of the posterior (caudal) surface of the bone. Each bone was placed with the lateral surface facing up and the axis of the elbow joint directed vertically. The camera was mounted pointing downwards and the height adjusted to 1.75 times bone length. A centimetre scale was placed beside the bone. The images were prepared in PowerPoint by overlaying a line representing the chord with 13 equally-spaced lines crossing it perpendicularly (Richmond & Whalen, 2001). For the ulna, the first intersection was placed on the posterior margin of the shaft in line with the coronoid process, and the thirteenth intersection on the posterior margin at the distal point of minimal circumference. For the humerus, the first intersection was placed on the posterior margin just distal to the head, and the thirteenth intersection on the posterior margin at the top of the olecranon fossa. The images were then transferred to tpsDig using tpsUtil software (Rohlf, 2015) and the points (constructed landmarks) where the thirteen lines intersected the posterior margin were digitised (Fig. 1).

The 2D coordinate data were translated, scaled and rotated so that the first constructed landmark was at the origin (0, 0) and the distal constructed landmark had the coordinate (1, 0) (Bookstein, 1986; Richmond & Whalen, 2001). The largest *y*-value among the constructed landmarks represented the normalised curvature (= max subtense/chord length). ANOVA was used to test for curvature differences among the locomotor categories (1 = arboreal, 2 = semiarboreal, 3 = terrestrial). The species mean normalised curvature values were regressed against the species body mass estimates (Nowak, 1991). To determine if larger species have more or less curvature, this regression was performed for the absolute value of the normalised curvature as well as the positive and negative curvatures separately.

The normalised curvature provides a single value that best describes the overall curvature. Negative values are cranial and positive values are caudal curves. However, normalised curvature does not fully describe the sagittal curvature of the bone. For example, some bones have a caudal curvature proximally and a cranial curvature distally, and a single normalised curvature value does not adequately describe the shape of the bone shaft. Geometric morphometric analysis of the shape represented by the 13 two-dimensional landmarks can provide a more detailed analysis of the variation in curvature.

The coordinates of the 13 semilandmarks representing the curve for each specimen were submitted to geometric morphometric analysis (GMA) in morphologika (O’Higgins & Jones, 1998). The data were Procrustes registered, with the “enable reflections” option unticked, and then the data were submitted to principal components analysis. A multivariate regression of all PCs against locomotor category (1 = arboreal, 2 = semi-arboreal, 3 = terrestrial) was performed to determine if there were significant differences in shape between arboreal and terrestrial species.

## Results

**Figure 2:**
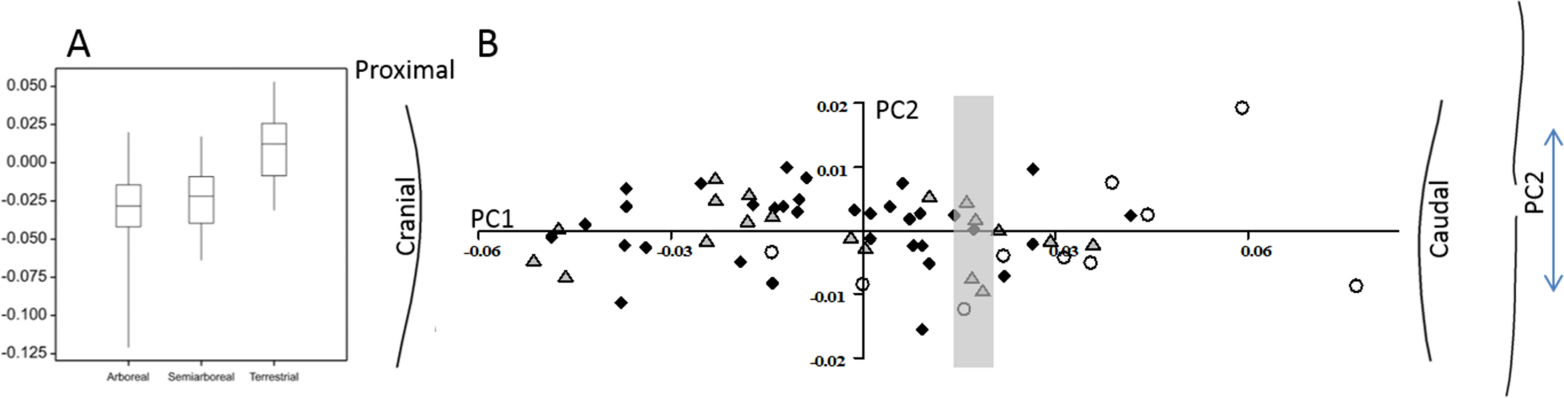
Primate ulnar curvature. (A) The overall curvature for the locomotor groups. (B) PC1 and PC2 from a geometric morphometric analysis of ulnar curvature. Curved lines represent the extremes of PC1 and PC2. The grey zone represents neutral curvature. Arboreal, black diamonds; semiarboreal, grey triangles; terrestrial, open circles.

Figure 2A shows the results for the primate ulna where there is a significant difference (*p* < 0.001) in the normalised curvature between arboreal and terrestrial species. The terrestrial species have caudally curved ulnae and the arboreal species have cranially curved ulnae. There was no relationship between body mass and curvature. Among arboreal species, the orangutan has the most negatively curved ulna and the galago has a positive (caudal) curvature. The gorilla (terrestrial) and the chimpanzees (semi-arboreal) with negative (cranial) curvatures are outliers from their respective groups.

The GM analysis of primate ulnar curvature shows that PC1 accounts for 85.5% of the total variation and codes for cranial versus caudal curvature of the ulna (Fig. 2B). Arboreal and semiarboreal species have significantly lower scores on PC1 than terrestrial species (both *p* < 0.005). Arboreal and semiarboreal species are not significantly different on PC1. PC2 accounts for 4.2% of the variation and codes for variations where some ulnae are S-shaped (caudal curvature proximally and cranial curvature distally), but this PC does not distinguish the locomotor categories. A multivariate regression of all the PCs on locomotor category is significant (*p* < 0.005) and explains 14.7% of the sample variance.

**Figure 3:**
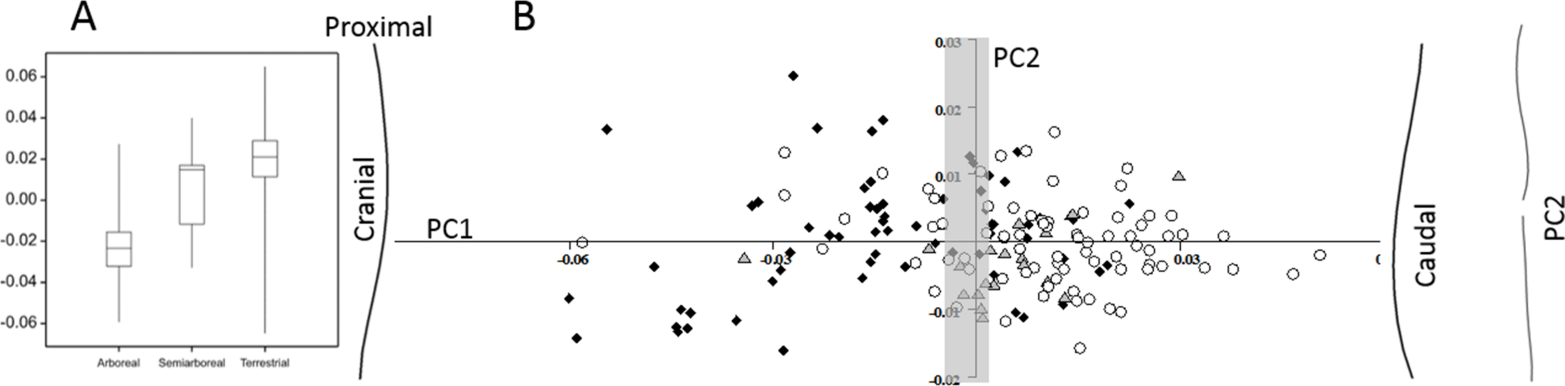
Marsupial ulnar curvature. (A) The overall curvature for the locomotor groups. (B) PC1 and PC2 from a geometric morphometric analysis of ulnar curvature. Curved lines represent the extremes of PC1 and PC2. The grey zone represents neutral curvature. Arboreal, black diamonds; semiarboreal, grey triangles; terrestrial, open circles.

For the marsupial ulna there is a difference (*p* < 0.001) in the normalised curvature between arboreal and terrestrial species (Fig. 3A). The terrestrial species have caudally curved ulnae and the arboreal species have cranially curved ulnae. There is no relationship between body size and degree of curvature. The *Phalanger gymnotis* (ground cuscus)—classified as terrestrial because it nests in burrows—is an outlier with a cranial ulna curvature. The tiny arboreal *Phascogale tapoatafa* registers a caudal curvature, but actually has an S-shaped ulna. In the GM analysis of the marsupial ulnar curvature (Fig. 2B), PC1 accounts for 78.9% of the shape variation and codes for cranial versus caudal curvature. Arboreal species have lower scores than semiarboreal or terrestrial species (*p* < 0.001 and *p* < 0.0001 respectively). Terrestrial and semiarboreal species are not different on PC1. PC2 accounts for 9.5% of the variation, and codes for straight versus S-shaped bones, but this PC does not distinguish arboreal from terrestrial species. A multivariate regression of all the PCs on locomotor category is significant (*p* < 0.0001) and explains 23% of the sample variance.

**Figure 4:**
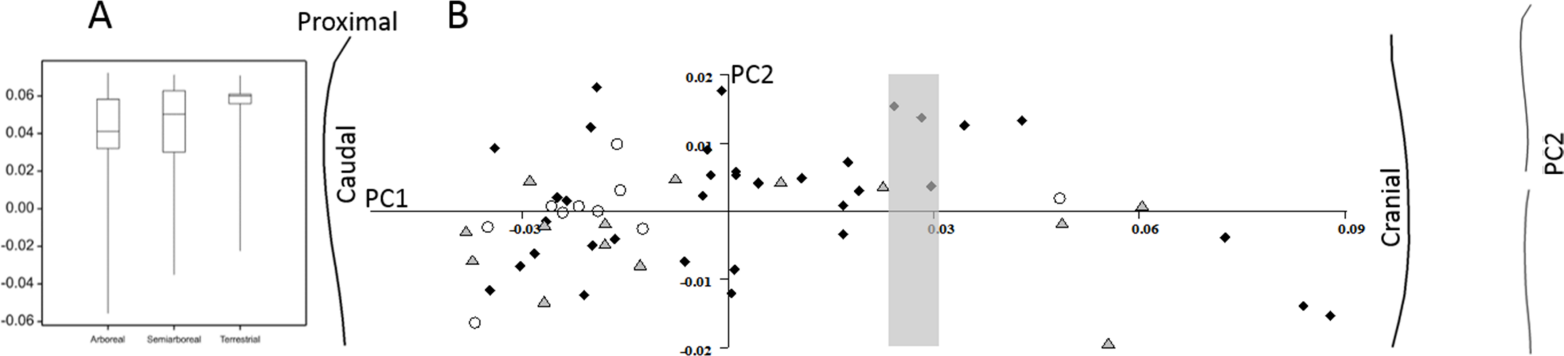
Primate humeral curvature. (A) The overall curvature for the locomotor groups. (B) PC1 and PC2 from a geometric morphometric analysis of humeral curvature. Curved lines represent the extremes of PC1 and PC2. The grey zone represents neutral curvature. Arboreal, black diamonds; semiarboreal, grey triangles; terrestrial, open circles.

When primate humeral curvature values are plotted by locomotor group (Fig. 4A) there are no significant differences. Most of the species have caudally curved humeri, but in each locomotor category there are outliers with negative curvature scores. These outliers are the apes (arboreal orangutans, semiarboreal chimpanzees and a terrestrial gorilla) and an indri. There is a relationship between body size and degree of curvature: the absolute value of curvature decreases with body size (*f* = 6.37, *p* = 0.022), but this is not significant (and the slope reverses) if apes are removed. When considering only positive (caudal) curvatures there is an increase in curvature with body size (*f* = 4.97, *p* = 0.037), but this disappears if the large baboons and the mandrill that have a high leverage on the regression are removed.

In the GM analysis of primate humeral curvature (Fig. 4B) PC1 accounts for 86.8% of the shape variation and codes for caudal (low scores) versus cranial curvature. However, the apes and the indri have high scores (eight data points on the right hand side of the plot) on PC1. PC2 accounts for 6.4% of the variation, but none of the PCs significantly distinguish locomotor groups.

**Figure 5:**
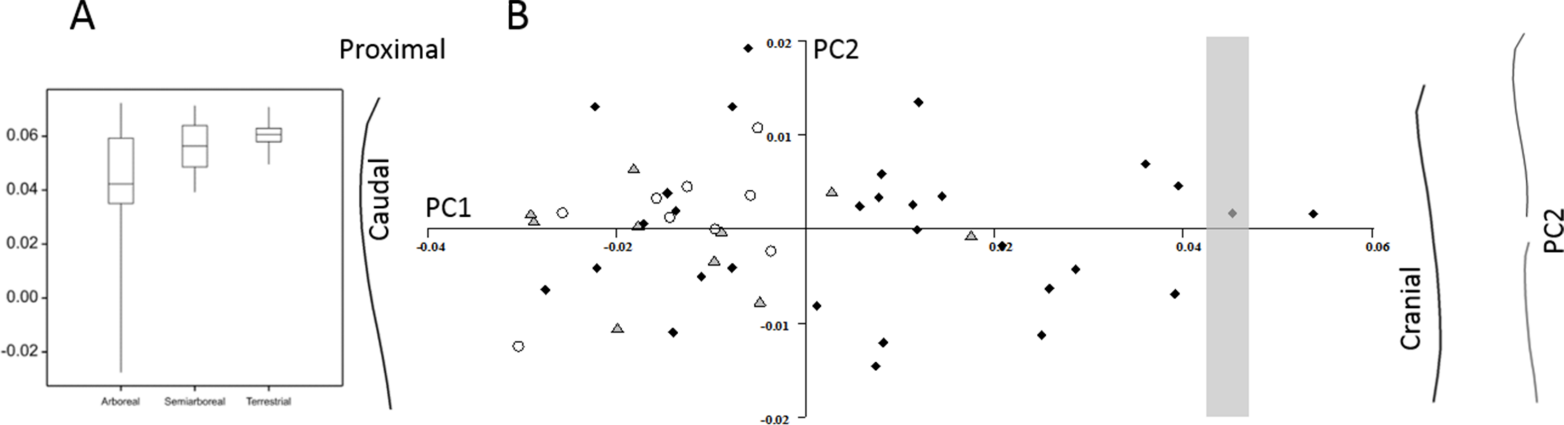
Primate humeral curvature (no apes). (A) The overall curvature for the locomotor groups. (B) PC1 and PC2 from a geometric morphometric analysis of humeral curvature. Curved lines represent the extremes of PC1 and PC2. The grey zone represents neutral curvature. Arboreal, black diamonds; semiarboreal, grey triangles; terrestrial, open circles.

When the apes are removed from the analysis the differences between the arboreal and semiarboreal (*p* < 0.05) and arboreal and terrestrial (*p* < 0.001) specimens become significant (see Fig. 5A). The remaining specimen with a positive curvature is the indri. In the GM analysis of primate humeral curvature, excluding apes (Fig. 5B), PC1 accounts for 77.2% of the variation and distinguishes between pure caudal curvature (to the left) and S-shaped curvature, with a cranial curve distally (to the right). PC1 in this analysis separates the arboreal species from semiarboreal (*p* < 0.05) and terrestrial (*p* < 0.01) species. PC2 accounts for 9.1% of the variation, but it and subsequent PCs do not distinguish locomotor groups. A multivariate regression of all the PCs against locomotor category is significant (*P* < 0.05) and explains 16.3% of the sample variance.

**Figure 6:**
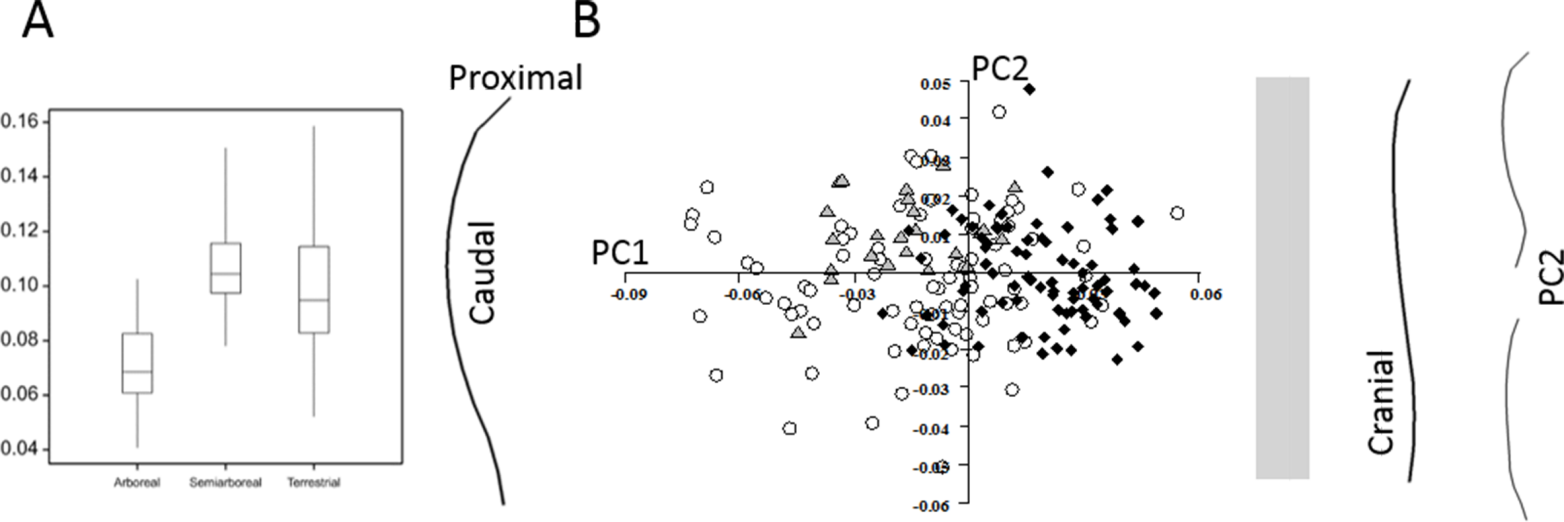
Marsupial humeral curvature. (A) The overall curvature for the locomotor groups. (B) PC1 and PC2 from a geometric morphometric analysis of humeral curvature. Curved lines represent the extremes of PC1 and PC2. The grey zone represents neutral curvature. Arboreal, black diamonds; semiarboreal, grey triangles; terrestrial, open circles.

When marsupial humeral curvature values are plotted by locomotor group (Fig. 6A) the terrestrial and semiarboreal species have more caudally-curved humeri than the arboreal species (ANOVA *p* < 0.001). Again, it should be noted that all the humeri have overall caudal curvatures in this analysis. There is no relationship between body size and curvature. In the GM analysis (Fig. 6B), PC1 accounts for 62.7% of the shape variation in marsupial humeri, and codes for a pure caudal curvature (to the left) to an S-shaped curve where the overall caudal curve is reduced and the distal end has a cranial curve (to the right). PC1 separates the arboreal from the semiarboreal (*p* < 0.0001) and terrestrial species (*p* < 0.0001). PC2 in this analysis accounts for 19.2% of the variation, and distinguishes the semiarboreal quolls from both the arboreal (*p* < 0.001) and terrestrial (*p* < 0.001) species. These quoll humeri are characterised by a marked cranial curvature distally with no reduction the proximal caudal curve. A multivariate regression of all the PCs against locomotor category explains 17.7% of the sample variance (*p* < 0.0001).

All the above analyses—including the normalised curvature and the GM analyses of curvature—were repeated using species means and the resulting trends were the same. All results remained significant among the marsupials, where there are more species in each category. However among the primates, where there are only four terrestrial species, the ANOVAs and multivariate regressions failed to reach significance. The differences in PC1 scores between terrestrial and arboreal species remained significant, and when the apes were removed from the ulna analyses the ANOVAs became significant.

## Discussion

The results broadly support the hypotheses and expectations of this study. For the ulna, terrestrial species tended to have caudally and arboreal species cranially curved bones. This supports the underlying idea that the ulna is curved in the opposite direction to the bending strains induced by the primary locomotor muscle action (habitual loading). Terrestrial species rely on elbow extension (triceps) to maintain stance against gravity, while arboreal species rely on elbow flexion (brachialis and brachioradialis) to climb and cling in the branches. For the humerus, differences in curvature between arboreal and terrestrial species were detected among both marsupials and primates. This suggests that the relationship between long bone curvature and habitual loading may be general and not restricted to the ulna.

### Ulnar curvature

**Figure 7:**
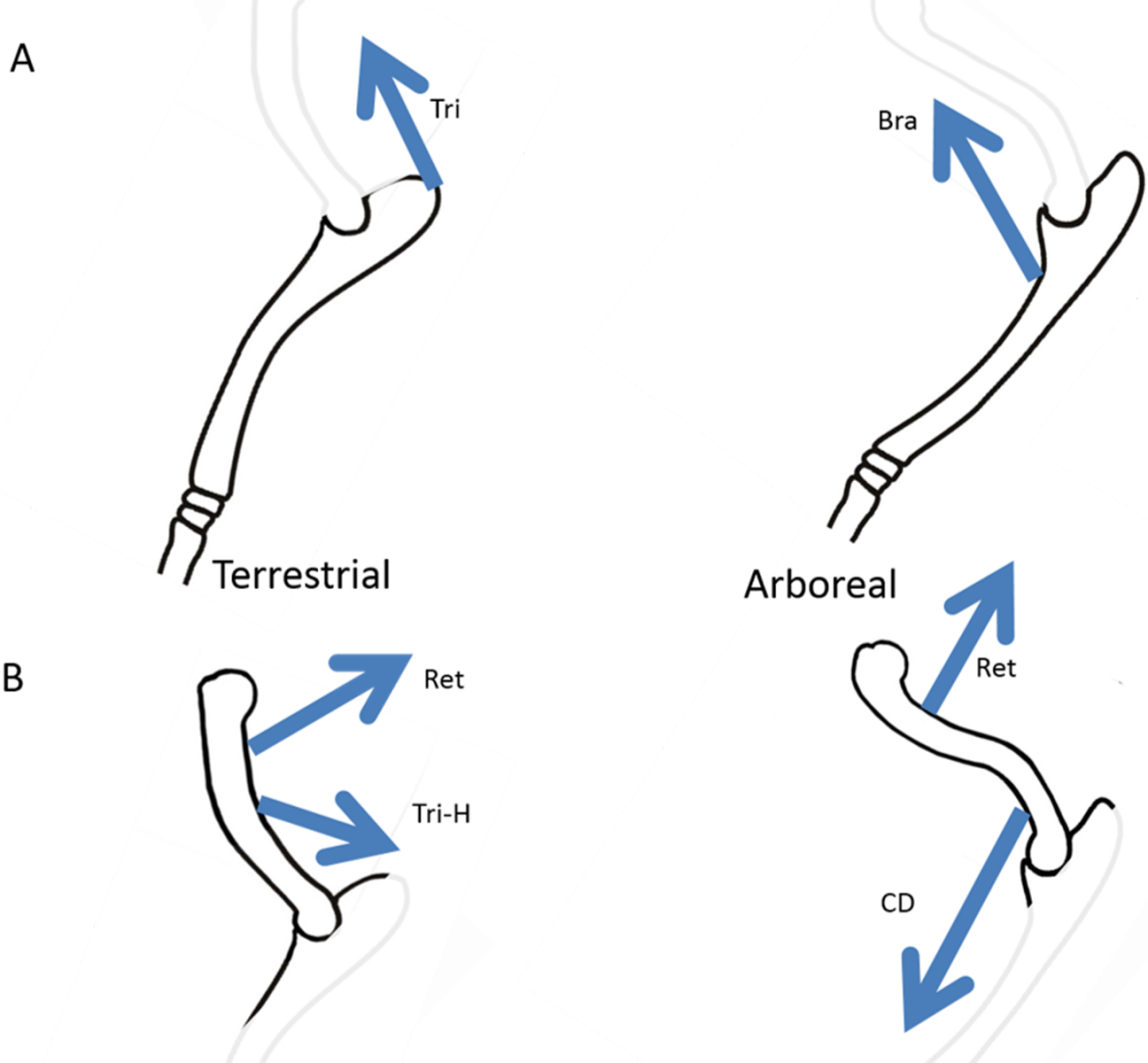
Habitual muscle loads thought to shape the ulnar and humeral curvature. (A) shows the typical curvatures of the ulna in terrestrial and arboreal species. (B) shoes the typical curvatures of the humerus in terrestrial and arboreal species. In each case the muscles proposed to be habitually active are indicated: Tri, triceps; Bra, brachialis; Ret, forelimb retractors; Tri-H, triceps humeral heads; CD, carpal and digital flexors and extensors (The length of the arrows is of no significance).

The differences between the curvature of the ulna among arboreal and terrestrial species is similar in primates and marsupials, and agrees with the original hypothesis (see Fig. 7). In terrestrial species of both groups, where the triceps muscle is required to maintain elbow extension to support the weight of the anterior part of the animal, the ulna has a caudal curvature. The action of triceps places the ulna under cranial bending strain, and this is countered by caudal strains arising from longitudinal forces acting on the caudally curved bone. On the other hand, in arboreal species of both groups, where the action of brachialis is required to support and raise the animal among the branches, the ulna has a cranial curvature. The action of brachialis induces caudal bending strains in the ulna, and this is countered by cranial bending strains from longitudinal forces acting on the cranially curved bone. In both the arboreal and terrestrial species, these longitudinal forces are due to the action of carpal and digital flexors and extensors which arise from the humerus or proximal radius and ulna. Among terrestrial species, the ground reaction forces would also contribute to this overall longitudinal compression.

### Humeral Curvature

This study found differences in humeral curvature between terrestrial and arboreal species, and these differences were consistent between the primates and the marsupials (see Fig. 7). Terrestrial species tended to have humeri curved uniformly in the caudal direction. In arboreal species, this caudal curvature was reduced and restricted to the proximal humerus, and the distal humerus showed cranial concavity. According to the curved bone theory presented here (and seen clearly in the ulna), we would expect that the caudal curvature in the humerus is an adaptation to resist habitual locomotor muscle action producing cranial bending in that bone. The muscles most likely to cranially bend these terrestrial humeri are the humeral heads of triceps, and shoulder muscles that pull caudally on the humeral shaft (eg. posterior deltoid, teres major and latissimus dorsi). The humeral heads of triceps are obviously active throughout stance phase, but the shoulder muscles are not involved in maintaining stance; instead they are used to propel the terrestrial animal forward (Harrison et al., 2012).

In the case of arboreal species (both primate and marsupial) the humerus is cranially curved (concave on the cranial side) in its distal half. The curved bone theory would suggest that this is a response to muscles pulling cranially in that region. It seems clear that these muscles would be carpal and digital flexors, the corresponding extensors, and brachioradialis, all of which have attachments to the distal humerus (supracondylar ridges) and produce a cranial pull (see Fig. 7) (Taylor, 1974; Evans, 1993; Youlatos, 2000). These muscles are active in climbing, clinging, and grasping. Meanwhile, the caudal curve in the proximal humeri of arboreal species could be an adaptation to the habitual action of forelimb extensors (posterior deltoid, teres major and latissimus dorsi) (Taylor, 1974; Evans, 1993; Youlatos, 2000), which are also necessary for climbing and clinging.

It is unclear why the apes stand apart from the other primates in their pattern of humeral curvature. It seems unlikely that this could be a phylogenetic trait unrelated to their behaviour or limb loading. All apes are highly arboreal as infants and subadults, so their seemingly-arboreal pattern of humeral curvature could arise from behaviours during development and growth. But even this seems unlikely, as it would be risky for adults to retain a curved bone that is not adaptive for their current habitual activities. An alternative explanation could be that gorillas and chimps employ knuckle walking as adults; perhaps this unique locomotive behaviour is somehow responsible for the distinct humeral morphology seen in the apes here.

The presence of the apes in the primate sample is not the only reason that the curvature results are not as clear-cut in the primate ulna and humerus. The sample size of primate specimens was low, and this meant that some clear differences did not reach significance. Among the primates there very few species that are truly terrestrial, even the baboons readily climb trees. Further the ulnar and humeral curvature of semi arboreal species are not clearly distinguished from the arboreal primates. On the other hand, among marsupials there are many species that are highly terrestrial, some that never enter the trees, some can climb and some dig burrows. There also many fully arboreal marsupial species, but there are fewer that are classified as semiarboreal and their forelimb bone shape is not well distinguished from that of the terrestrial species.

This study examined forelimb bone curvature in primates and marsupials—two groups of mammals that contain arboreal as well as terrestrial species. Such widely separated groups of mammals were chosen because, if similar variation in curvature accompanies arboreal and terrestrial locomotor styles in both groups, then it cannot be argued that the curvature observed in the forelimb bones of arboreal and terrestrial species is an inherited characteristic. Further, examination of Tables 1 and 2 shows that locomotor categories are not segregated by family, but spread across phylogeny.

Other groups of mammals also contain species with arboreal and terrestrial habits; notably, rodents are very diverse. A study by Candela & Picasso (2008) examined a range of extant rodent species to gain a better understanding of the behaviour of Miocene porcupines. While Candela & Picasso (2008) do not analyse curvature, their Fig. 8 (page 562) shows a selection of rodent ulnae in lateral view. The arboreal coendou has a clear cranial curvature, while the terrestrial mara, cavi, vizcacha and chinchilla all have caudal ulna curvature. So it seems highly likely that, at least for the ulna, the pattern observed in the present study will also be seen in rodents and, potentially, other groups of mammals. The present study is a preliminary one to demonstrate the idea that limb bone curvature is related to habitual loading. Further studies could be performed using much larger samples of primates and also examining other groups of mammals such as rodents.

## Conclusions

This study has demonstrated that the curvature of the ulna and humerus is qualitatively different in arboreal and terrestrial species. Arboreal and terrestrial lifestyles place quite different demands on the forelimb bones, and this is clearly reflected in their curvature. This strongly supports the idea that long bone curvature is an adaptation to habitual loading (Milne, 2016). This study raises the question of the mechanism by which these bones adapt to their habitual loading. If, as suggested by Frost (1964), bone tissue tends to be deposited on surfaces under compression and resorbed from surfaces under tension, then the observed curvature could be caused by the habitual loading. For example, in terrestrial mammals the habitual action of triceps places cranial bending strains on the ulna (compression on the cranial side and tension on the caudal side), which causes them to become caudally curved, or concave. The opposite strains would be imposed on the ulna of arboreal species, and we would likewise expect the ulna to develop the opposite curvature. As curvature develops, longitudinal forces acting through the bone begin to generate opposing bending strains that reduce or cancel the strains due to habitual muscle action (see Frost, 1964; Figs. 5 and 7). Equilibrium would be reached, and the level and direction of curvature would stabilise, thus giving rise to the curvatures observed in mammalian limb bones.

##  Supplemental Information

10.7717/peerj.3229/supp-1 Supplemental Information 1 Marsupial humerus coordinates

10.7717/peerj.3229/supp-2 Supplemental Information 2 Marsupial humerus specimen data

10.7717/peerj.3229/supp-3 Supplemental Information 3 Marsupial ulna coordinates

10.7717/peerj.3229/supp-4 Supplemental Information 4 Marsupial ulna specimen data

10.7717/peerj.3229/supp-5 Supplemental Information 5 Primate humerus coordinates

10.7717/peerj.3229/supp-6 Supplemental Information 6 Primate humerus specimen data

10.7717/peerj.3229/supp-7 Supplemental Information 7 Primate ulna coordinates

10.7717/peerj.3229/supp-8 Supplemental Information 8 Primate ulna specimen data

## Additional Information and Declarations

### Data Availability

The following information was supplied regarding data availability: The raw data has been supplied as a Supplementary File.
